# A Pilot Clinical Study of Liquid Ubiquinol Supplementation on Cardiac Function in Pediatric Dilated Cardiomyopathy

**DOI:** 10.3390/nu10111697

**Published:** 2018-11-07

**Authors:** Fong-Lin Chen, Po-Sheng Chang, Yi-Chin Lin, Ping-Ting Lin

**Affiliations:** 1 Department of Pediatrics, Chung Shan Medical University Hospital, Taichung 40201, Taiwan; cshy053@gmail.com; 2 School of Medicine, Chung Shan Medical University, Taichung 40201, Taiwan; 3 Pediatric Cardiology Clinic, Taichung 40244, Taiwan; 4 Department of Nutrition, Chung Shan Medical University, Taichung 40201, Taiwan; ccrazybon@gmail.com (P.-S.C.); ymlin@csmu.edu.tw (Y.-C.L.); 5 Graduate Program in Nutrition, Chung Shan Medical University, Taichung 40201, Taiwan; 6 Department of Nutrition, Chung Shan Medical University Hospital, Taichung 40201, Taiwan

**Keywords:** liquid ubiquinol, coenzyme Q10, pediatric dilated cardiomyopathy, heart failure, pediatric nutrition, dietary supplementation

## Abstract

Background: Pediatric dilated cardiomyopathy (PDCM) is a life-threatening type of cardiac muscle dysfunction in children. Ubiquinone is a lipid-soluble nutrient that participates in energy synthesis. Recently, a novel hydrophilic ubiquinol supplement was developed. The purpose of this study was to assess the effect of liquid ubiquinol supplementation (10 mg/kg body weight/day) on cardiac function in children with PDCM. Methods: Ten children diagnosed with PDCM were recruited to this study and administered with liquid ubiquinol for 24 weeks. The cardiac function was measured by echocardiography. The New York Heart Association (NYHA) functional classification was used to assess symptoms of heart failure. Plasma coenzyme Q10 levels were measured during the study. Results: Ejection fraction (EF) and fractional shortening (FS) were significantly higher than the baseline values until week 16 of supplementation. Subjects who had higher plasma coenzyme Q10 concentration had significantly better EF and FS values. In addition, 30% of the subjects showed improvement in the NYHA classification after 24 weeks of supplementation. Conclusion: Liquid ubiquinol supplementation is associated with an increase the level of coenzyme Q10 to complementary improve cardiac function (particularly EF and FS) and ameliorate the symptoms of heart failure in children with PDCM.

## 1. Introduction

Pediatric dilated cardiomyopathy (PDCM) is the most common primary cardiomyopathy. Approximately 60% of pediatric cardiomyopathy cases involve dilated cardiomyopathy, which is a serious disorder of cardiac muscle that progresses rapidly to death in children [1,2,3]. The causes of PDCM may be idiopathic myocarditis (16%), neuromuscular dysfunction (9%), familial dilated cardiomyopathy (5%), inborn errors of metabolism (4%) or malformation syndrome (1%); most etiologies (75%) are unknown [4]. Most children with PDCM are treated with drugs (such as digitalis, diuretics, or angiotensin-converting enzyme inhibitors) that may relieve the symptoms of heart failure and maximize cardiac function [5]. However, such medical therapy is sometimes not effective in cases where the disease progresses rapidly, and cardiac transplantation is the final conventional therapy for PDCM [6,7]. Most children with PDCM face life-threatening situations due to the paucity of heart donors [8]. Thus, using nutritional complementary therapy to improve cardiac function could be considered a therapeutic strategy in children with PDCM.

Coenzyme Q10 is a lipid-soluble nutrient component that participates in the mitochondrial respiratory chain of adenosine triphosphate (ATP) synthesis [9,10]. Most coenzyme Q10 exists in the form of ubiquinone (an oxidized form of coenzyme Q10) in capsule/tablet supplements. After oral ingestion, ubiquinone may be transformed to ubiquinol (a reduced form of coenzyme Q10), which has physiological functions in the body [11]. Recently, Mae et al. [12] developed a hydrophilic ubiquinol supplement (liquid ubiquinol). This solubilized formulation of ubiquinol seems to have a superior bioavailability [13]. To date, few studies have been conducted to investigate coenzyme Q10 supplementation in children with PDCM. Only three clinical studies have investigated the treatment of cardiac failure in PDCM with an oxidized capsule form of coenzyme Q10 (ubiquinone) [14,15,16]. However, in clinical practice, children may not be able to easily ingest capsule or tablet forms of coenzyme Q10. In addition, hydrophilic coenzyme Q10 supplements seem exhibit a higher uptake and absorption than lipophilic forms, as demonstrated in cellular and human studies [17,18]. It is worth trying to use a liquid ubiquinol supplement to understand its impact on cardiac function in children with PDCM. Thus, the purpose of this study was to assess the effect of liquid ubiquinol supplementation (10 mg/kg body weight) on cardiac function in children with PDCM.

## 2. Materials and Methods

### 2.1. Study Design and Subjects

This clinical study was conducted as an open labeled trial. Children with PDCM (age ≤ 20 years) and ejection fractions (EF) of ≤40% were measured by echocardiography and diagnosed by a cardiologist. We excluded children with hypertension, acute myocarditis, or current use of antioxidant supplements, coenzyme Q10 supplements, or warfarin therapy; pregnant teenagers were also excluded. The study was approved by the Institutional Review Board of Chung Shan Medical University Hospital, Taiwan, and the clinical trial was registered at Clinical Trials. gov (NCT02847585). This clinical trial started recruiting subjects in August 2016, and data acquisition for the last subject was completed in December 2017. Each subject or his/her legal representative provided written informed consent to participate in the study.

### 2.2. Intervention

A total of 10 PDCM children were recruited to this study and assigned to the liquid ubiquinol (QuinoMitQ10^®^ Fluid, MSE Pharmazeutika GmbH, Bad Homburg, Germany, 10 mg/kg/day) supplementation. The dose of the supplementation was according to the previous study [14]. The intervention was administered for 24 weeks. Before the study, the investigators instructed the subjects to take the liquid ubiquinol supplements before meals. Every drop of liquid supplement provided 8.3 mg of ubiquinol. The investigators instructed the subjects to take the supplement daily according to their current body weight. To monitor the compliance of the subjects, the investigators asked the subjects to return the supplied bottle of supplement every four weeks, and weighed the supplement bottle to verify its usage by the subjects.

### 2.3. Anthropometric and Hematologic Measurements

Characteristic data of each subject were acquired using questionnaire and medical records. The subjects’ anthropometric data, such as body weight, height, and head and mid-upper circumferences were measured, and body mass index (kg/m^2^) was calculated. Blood specimens were collected in vacutainer tubes (Becton Dickinson, Rutherford, NJ, USA) containing EDTA (ethylenediaminetetraacetic acid) and no anticoagulants as required at weeks 0, 12, and 24. Plasma and serum were prepared after centrifugation (3000 rpm, 4 °C for 15 min), and then stored at −80 °C until analysis. Creatine kinase (CK) and creatine kinase-muscle/brain (CK-MB) were analyzed by using an automated biochemical analyzer (Hitachi 7070 & 7600; Hitachi High-Technologies Corporation, Tokyo, Japan). B-type natriuretic peptide (BNP) and N-terminal pro BNP (NT-pro BNP) were analyzed by using an automated immunoanalyzer (Abbott ARCHITECT i2000 SR and bioMérieux). Plasma coenzyme Q10 levels were measured by using high-performance liquid chromatography [19].

### 2.4. Assessments of Cardiac Function and Symptoms of Heart Failure

Cardiac function was measured by echocardiography by using a Color-Doppler-echocardiographic machine (PHILIPS iE33, Amsterdam, The Netherlands). EF, fractional shortening (FS), end-diastolic volume (EDV), end-systolic volume (ESV), left ventricular internal diameter end-diastole (LVIDd), and left ventricular internal diameter end-systole (LVIDs) were measured by M-mode. Left ventricular outflow tract diameter (LVOTd), stroke volume (SV), and heart rate (HR) were measured by pulsed Doppler. The cardiac output (CO), cardiac index (CI), and myocardial performance index (MPI) were calculated using the following formulas: CO = SV × HR; CI = CO ÷ body surface area; MPI = (isovolumetric contraction time (ICT) + isovolumetric relaxation time (IRT)) ÷ ejection time (ET). Symptoms of heart failure were measured by using the New York Heart Association (NYHA) functional classification: Class I: no limitation of physical activity, ordinary physical activity does not cause undue fatigue, palpitations, or dyspnea; Class II: slight limitation of physical activity, comfortable at rest, ordinary physical activity results in fatigue, palpitation, and dyspnea; Class III: obvious limitation of physical activity, comfortable at rest, less than ordinary activity causes fatigue, palpitation, or dyspnea; Class IV: unable to perform any physical activity without discomfort, symptoms of heart failure at rest.

### 2.5. Statistical Analysis

All statistical analyses were performed using SigmaPlot software (version 12.0, Systat, San Jose, California, CA, USA). The Shapiro–Wilk test was used to examine the normal distribution of variables. One-way repeated measures ANOVA or Friedman repeated measure ANOVA on ranks was used to compare the mean values of continuous variables at baseline (week 0) and at weeks 12 and 24 within groups, and *post hoc* tests were used to further examine the significance of differences within groups. McNemar’s tests were used to compare the percentage of subjects at each NYHA classification after intervention. A paired *t*-test or Wilcoxon signed-rank test was used to compare differences within groups between two time points. Spearman’s rank order correlation coefficient was used to evaluate the correlation between the plasma coenzyme Q10 level and cardiac function after supplementation for 12 weeks and 24 weeks. The values presented in the text are the means ± standard deviations, as well as the medians. Statistical results were considered to be significant at a *p* value of ≤ 0.05.

## 3. Results

### 3.1. Characteristics of the Subjects

The baseline characteristics of the subjects are shown in Table 1. The mean age of the subjects was 10 years, and the gender ratio of boys to girls was 1:1. The mean dose of the liquid ubiquinol supplement was at 350 mg/d (range 110–700 mg/d). With regard to medications, 40% of the subjects used angiotensin II receptor antagonists, angiotensin-converting enzyme inhibitors, loop diuretics, and potassium-sparing diuretics, and 10% of the subjects were under beta-blocker therapy, and 30% of the subjects were under digoxin therapy.

### 3.2. Cardiac Function and Symptoms of Heart Failure after Supplementation

The mean level of plasma coenzyme Q10 was 0.43 ± 0.12 μM at baseline, which significantly increased to 3.90 ± 1.45 μM after 24 weeks of supplementation (*p* < 0.01). Table 2 shows the levels of cardiac function after supplementation. In the echocardiographic data, EF (*p* = 0.15) and FS (*p* = 0.19) were slightly increased, and the ESV was slightly decreased (*p* = 0.15) after 24 weeks of supplementation. There was no significant difference in the cardiac-related hematologic data after supplementation. 

Table 3 shows the correlations between plasma coenzyme Q10 level and cardiac function after supplementation. The plasma coenzyme Q10 concentration was slightly moderate positively correlated with EF (*r* = 0.48, *p* = 0.15) and FS (*r* = 0.37, *p* = 0.28) after 12 weeks of supplementation; however, the correlations between plasma coenzyme Q10 and EF or EF became slightly weaker after 24 weeks of the supplementation (EF, *r* = 0.37, *p* = 0.28; FS, *r* = 0.26, *p* = 0.45). In addition, the plasma coenzyme Q10 concentration was slightly moderate negatively correlated with CK-MB activity (*r* = −0.48, *p* = 0.17) after 12 weeks of supplementation, and showed a significantly high negatively correlated with CK activity (*r* = −0.75, *p* < 0.05) after 24 weeks of supplementation.

**Figure 1:**
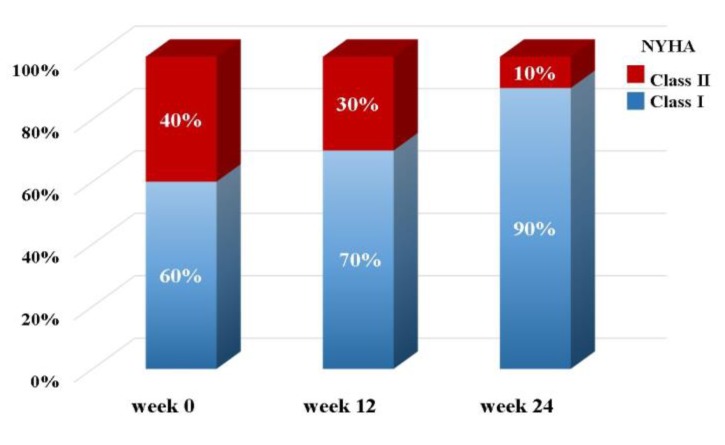
The percentages of subjects at each New York Heart Association (NYHA) functional classification after supplementation.

The percentage of subjects at each NYHA functional classification after supplementation are shown in Figure 1. Forty percent of the subjects were in NYHA Class II at baseline (week 0), and the percentage of subjects categorized in NYHA Class II decreased to 10% (*p* = 0.25) after 24 weeks of supplementation.

**Figure 2:**
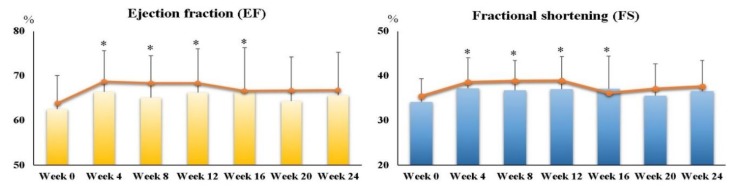
Ejection fraction (EF) and fractional shortening (FS) in children with PDCM after every four weeks of liquid ubiquinol supplementation. ∙ Median values. * Values were significantly different from those at week 0 (*p* ≤ 0.05). EF, ejection fractions; FS, fractional shortening; PDCM, pediatric dilated cardiomyopathy.

Furthermore, we examined EF and FS after every 4 weeks of supplementation, and the data are shown in Figure 2. Both EF and FS at week 4 (EF, *p* < 0.01; FS, *p* < 0.01), week 8 (EF, *p* = 0.05; FS, *p* = 0.02), week 12 (EF, *p* = 0.04; FS, *p* = 0.05), and week 16 (EF, *p* = 0.04; FS, *p* = 0.04) were significantly higher than those at week 0. 

**Figure 3:**
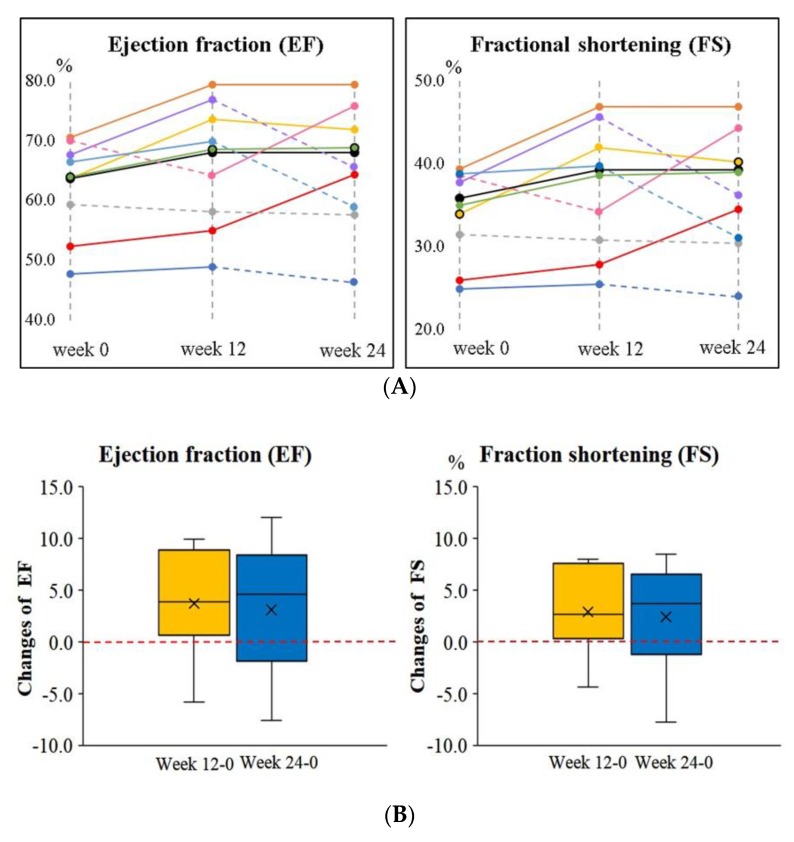
Changes in ejection fraction (EF) and fractional shortening (FS) in children with PDCM after supplementation. (**A**) EF and FS for each subject. The solid line indicates a rise, and the dotted line indicates a decline during the intervention. (**B**) The changes in EF and FS between week 12 and week 24. EF, ejection fractions; FS, fractional shortening; PDCM, pediatric dilated cardiomyopathy.

The changes in EF and FS for each subject after supplementation are shown in Figure 3. Eighty percent and 60% of the subjects had increased EF and FS after 12 weeks and 24 weeks of supplementation, respectively. The mean change in EF was 3.72 ± 4.93%, and the mean change in FS was 2.88 ± 4.07% after 12 weeks of supplementation; the mean change in EF was 3.11 ± 6.10%, and the mean change in FS was 2.40 ± 5.10% after 24 weeks of supplementation.

**Figure 4:**
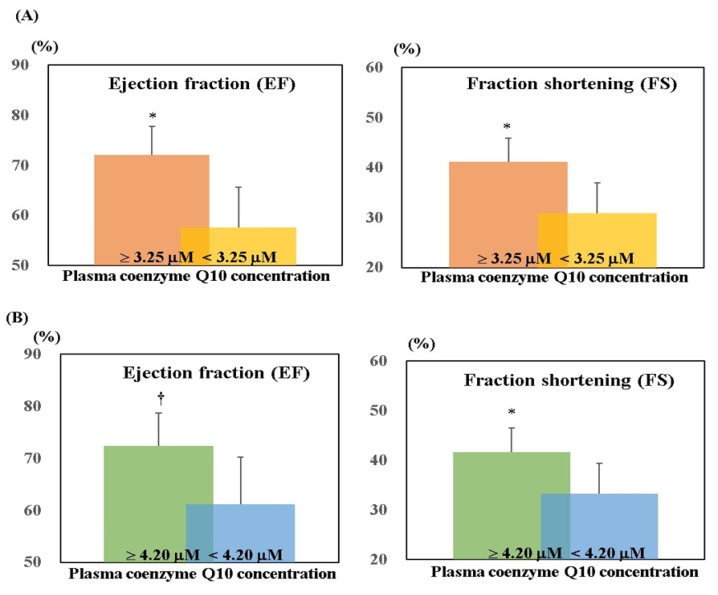
EF and FS according to the level of plasma coenzyme Q10. (**A**) At week 12. (**B**) At week 24. * *p* ≤ 0.05; † *p* = 0.07. EF, ejection fractions; FS, fractional shortening; PDCM, pediatric dilated cardiomyopathy.

Figure 4 shows EF and FS according to the level of plasma coenzyme Q10. After 12 weeks of supplementation, subjects with higher plasma coenzyme Q10 concentration (≥3.25 μM) had significantly higher EF (*p* < 0.01) and FS (*p* = 0.02) than those with lower plasma coenzyme Q10 concentration. After 24 weeks of supplementation, subjects with higher plasma coenzyme Q10 concentration (≥4.20 μM) had significantly higher EF (*p* = 0.07) and FS (*p* = 0.05) than those with lower plasma coenzyme Q10 concentration.

## 4. Discussion

Coenzyme Q10 supplementation can serve as a complementary therapy for adult heart failure, as has been well-demonstrated in many clinical studies [20,21,22,23]. The heart is the organ that contains the highest concentration of coenzyme Q10 in the human body [24]. Studies have revealed that adult patients with heart failure have a significantly lower level of coenzyme Q10 [25,26,27], and that a higher concentration of coenzyme Q10 in cardiomyocytes can complementary improve their cardiac contraction [11,28,29]. In pediatric practice, almost 80% of children with PDCM have a poor cardiac contraction due to congenital left ventricular dilatation, and this condition may progress to heart failure [30,31]. Few clinical studies have investigated the level of coenzyme Q10 in pediatric cases. Miles et al. [32] investigated 68 healthy children aged 0–18 years, and found that their mean level of coenzyme Q10 was 0.97 μM. Considering the report of Miles et al., it seems that the children with PDCM in the present study had a significantly lower level of coenzyme Q10 than the healthy children before the intervention (0.43 ± 0.12 μM). However, after 12 weeks and 24 weeks of liquid ubiquinol supplementation, the level of plasma coenzyme Q10 was increased significantly by 7.8 times and 9.4 times, respectively. The coenzyme Q10 deficiency was successfully adjusted after 12 weeks of liquid ubiquinol supplementation. An increasing level of coenzyme Q10 showed a significant correlation with the EF and FS (Figure 4), which indicated improved cardiac contraction in the children with PDCM. With regard to the hematologic values associated with myocardial cell injury and heart failure, such as CK, CK-MB, BNP, and NT-pro BNP [33,34], we detected a slight decrease in the level of NT-pro BNP after 24 weeks of supplementation (Table 2), but a highly significant negative correlation was found between plasma coenzyme Q10 and CK activity after 24 weeks of supplementation (Table 3). Additionally, we also noticed a slightly negative correlation between plasma coenzyme Q10 and CK-MB at 12 weeks and 24 weeks. CK-MB has been regarded as biochemical markers of myocyte necrosis, and the level may elevate due to the inflammation [34,35]. Coenzyme Q10 could be an anti-inflammatory nutrient by inhibiting the inflammatory cascade of NF-κB activation [36,37]. Therefore, liquid ubiquinol supplementation might yield an improvement in cardiac contraction through regulating the level of coenzyme Q10 to delay myocardial damage in PDCM.

Elshershari et al. [14] were the first investigators to try ubiquinone (an oxidized form of coenzyme Q10 with soybean oil, 10 mg/kg body weight/day) capsule supplement in children with PDCM. The researchers found that in six children with PDCM children, EF (41.0 ± 6.9% increased to 60.3 ± 10.7%, *p* < 0.01) and FS (17.3 ± 3.1% increased to 30.0 ± 5.2%, *p* < 0.01) were significantly increased from four weeks to 64 weeks of supplementation. Soongswang et al. [15] also treated children with PDCM with an ubiquinone capsule supplement, but the dose of the supplement was set at 3 mg/kg body weight/day. In that study, the researchers observed only a slight improvement in EF (30% increased to 37.5%, *p* = 0.15) after 36 weeks of supplementation [15]. A randomized, placebo-controlled study was conducted by Kocharian et al. [16], who treated children with PDCM with an oxidized coenzyme Q10 supplement (10 mg/kg body weight per day) for 24 weeks, and found that EF and FS were significantly increased (*p* < 0.01). Based on the aforementioned studies, coenzyme Q10 supplements should apparently be given at a dose of 10 mg/kg body weight for PDCM to yield a cardio protective impact on the heart [14,16]. In the present study, we tried using liquid ubiquinol at a dose of 10 mg/kg in PDCM children, and found that EF and FS significantly increased by 7–10% after 12 weeks of supplementation (Figure 2); this increase lasted until week 16. However, the increase in EF and FS become weaker (2–4%) at week 20 and week 24 (Figure 2). We also noted that the level of plasma coenzyme Q10 at week 12 should be higher than 3.25 μM in order to increase EF and FS significantly; however, the level of plasma coenzyme Q10 had to be higher than the level of 4.20 μM to exhibit this remarkable effect (Figure 4). In the present study, all of the subjects were under medical therapy, but their prescriptions were not changed during the study. In such a stable condition of the subjects, we still successfully detected a significantly improved impact on cardiac function after the intervention. Although these significant impacts of EF and FS became weaker at week 20 and week 24, both EF and FS were maintained, and did not decrease significantly below than the baseline values (Figure 2 and Figure 3). As a result, we suggest that liquid ubiquinol supplementation could be used as an adjunctive therapy for PDCM. 

In this study, we also assessed the symptoms of heart failure by the NYHA functional classification after supplementation. At baseline, 40% of the subjects were in NYHA Class II, and this value decreased to 10% after 24 weeks of liquid ubiquinol supplementation (Figure 1). In addition, we also observed that subjects with higher plasma coenzyme Q10 may have a significantly improved NYHA functional class in the present study (data not shown). After 24 weeks of supplementation, the changes of plasma coenzyme Q10 level in symptom-improving subjects was significantly higher than those without improvement (4.67 ± 1.25 μM versus 2.95 ± 1.20 μM, *p* = 0.07). As a result, we suggest that liquid ubiquinol supplementation can improve the symptoms of heart failure by increasing the coenzyme Q10 levels in children with PDCM.

The strength of this study is that it is the first clinical study to use liquid ubiquinol supplementation in children with PDCM. Moreover, this supplement is given in the form of an oral drop, which is easily to ingested by children, and thus can be used in clinical practice. Third, in this pilot study, we provided direct and complete evidence to clarify the relationship between the plasma coenzyme Q10 level and cardiac function. Although the intervention was investigated for up to 24 weeks (six months) in the present study, longer interventional studies with cross-over design should be performed, as they might be helpful to clarify the causality of the intervention. Recently, studies have indicated that pathogenic mutations may be involved in the biosynthesis of coenzyme Q10 [38,39]. Coenzyme Q10 biosynthesis may be affected by *COQ* genes’ mutations, and thus influence mitochondrial energy production in myocardial tissue [39,40]. Thus, further studies are necessary in order to investigate the pathogenesis of coenzyme Q10 deficiency in PDCM with different molecular defects, and subsequently develop more effective therapies.

## 5. Conclusions

In this clinical study, we suggest that liquid ubiquinol supplementation is associated with an increase in the level of coenzyme Q10 to complementary improve cardiac function (EF and FS) and ameliorate the symptoms of heart failure in children with PDCM.

## Figures and Tables

**Table 1:** Characteristics of the subjects.

	PDCM (*n* = 10)
Gender (n, boys/girls)	5/5
Age (y)	9.9 ± 5.8 (9.0) ^1^
Height (cm)	134.3 ± 28.8 (131.0)
Body weight (kg)	35.0 ± 20.0 (26.3)
Body mass index (kg/m^2^)	17.7 ± 3.3 (16.0)
Head circumference (cm)	51.7 ± 2.8 (51.0)
Mid-upper arm circumferences (cm)	19.0 ± 4.8 (17.0)
Dose of liquid ubiquinol (mg/d)	349.5 ± 200.4 (262.5)
**Medications (*n*, %)**	
Angiotensin II receptor antagonist	4 (40%)
Angiotensin-converting enzyme inhibitors	4 (40%)
Beta-blockers	1 (10%)
Digoxin	3 (30%)
Loop diuretics	4 (40%)
Potassium-sparing diuretics	4 (40%)

^1^ means ± standard deviations (medians). PDCM, pediatric dilated cardiomyopathy.

**Table 2:** Levels of cardiac function after liquid ubiquinol supplementation.

	Week 0	Week 12	Week 24	*p* Values
**Echocardiography**				
EF (%)	62.56 ± 7.47 (63.85) ^1^	66.28 ± 9.78 (68.35)	65.67 ± 9.63 (66.85)	0.15
FS (%)	34.20 ± 5.22 (35.45)	37.07 ± 7.28 (38.98)	36.59 ± 6.86 (37.60)	0.19
CO (L/min)	10.77 ± 14.47 (5.83)	16.36 ± 26.16 (5.72)	14.62 ± 26.18 (5.62)	0.73
SV (mL)	159.96 ± 274.25 (72.73)	176.32 ± 217.88 (84.10)	144.40 ± 217.56 (55.96)	0.44
HR (beat/min)	82.15 ± 27.82 (85.00)	82.6 ± 33.18 (83.75)	86.50 ± 27.07 (92.00)	0.93
LVOTd (cm)	1.75 ± 0.78 (1.60)	1.87 ± 0.61 (1.80)	1.68 ± 0.63 (1.65)	0.25
EDV (mL)	133.92 ± 104.69 (107.50)	132.75 ± 99.49 (109.00)	135.16 ± 98.41 (99.20)	0.93
ESV (mL)	53.12 ± 50.17 (38.05)	48.14 ± 46.70 (33.00)	51.05 ± 48.42 (41.45)	0.15
LVIDd (cm)	5.00 ± 1.61 (4.80)	4.99 ± 1.57 (4.83)	5.05 ± 1.53 (4.64)	0.89
LVIDs (cm)	3.32 ± 1.20 (3.10)	3.17 ± 1.18 (2.92)	3.25 ± 1.21 (3.22)	0.22
CI (L/(min*m^2^))	8.36 ± 8.61 (5.82)	12.15 ± 15.74 (5.60)	10.39 ± 15.71 (5.05)	0.73
MPI	0.84 ± 0.47 (0.66)	0.76 ± 0.34 (0.89)	0.75 ± 0.47 (0.70)	0.48
**Hematology**				
BNP (pg/mL)	115.6 ± 152.2 (56.9)	157.4 ± 212.1 (48.8)	148.0 ± 217.2 (50.8)	0.63
NT-Pro BNP (pg/mL)	357.1 ± 451.6 (236.0)	527.2 ± 703.8 (365.5)	458.3 ± 694.3 (191.5)	0.23
CK (U/L)	238.0 ± 344.0 (135.5)	436.2 ± 772.7 (141.5)	181.9 ± 105.5 (141.5)	0.70
CK-MB (U/L)	14.2 ± 6.65 (16.0)	10.8 ± 6.5 (12.0)	12.6 ± 8.4 (11.0)	0.52

^1^ means ± standard deviations (medians). BNP, B-type natriuretic peptide; CI, cardiac index; CK, creatine kinase; CK-MB, creatine kinase-muscle/brain; CO, cardiac output; EDV, end-diastolic volume; EF, ejection fractions; ESV, end-systolic volume; FS, fractional shortening; HR, heart rate; LVIDd, left ventricular internal diameter end-diastole; LVIDs; left ventricular internal diameter end-systole; LVOTd, left ventricular outflow tract diameter; MPI, myocardial performance index; NT-Pro BNP, N-terminal pro B-type natriuretic peptide; SV, stroke volume.

**Table 3:** Correlations between plasma coenzyme Q10 level and cardiac function after supplementation.

	Plasma Coenzyme Q10 Concentration (μM)	
	Week 12	Week 24
**EF (%)**	0.48 (0.15) ^1^	0.37 (0.28)
**FS (%)**	0.37 (0.28)	0.26 (0.45)
**CK (U/L)**	−0.33 (0.33)	−0.75 (<0.05)
**CK-MB (U/L)**	−0.48 (0.17)	−0.31 (0.37)

^1^*r*, correlation coefficients (*p* values). CK, creatine kinase; CK-MB, creatine kinase-MB; EF, ejection fractions; FS, fractional shortening.

## Abbreviations

ATP	adenosine triphosphate
BNP	B-type natriuretic peptide
CI	cardiac index
CK	creatine kinase
CK-MB	creatine kinase-muscle/brain
CO	cardiac output
EDV	end-diastolic volume
EF	ejection fractions
ESV	end-systolic volume
ET	ejection time
FS	fractional shortening
HR	heart rate
ICT	isovolumetric contraction time
IRT	isovolumetric relaxation time
LVIDd	left ventricular internal diameter end-diastole
LVIDs	left ventricular internal diameter end-systole
LVOTd	left ventricular outflow tract diameter
MPI	myocardial performance index
NT-pro BNP	N-terminal pro BNP
NYHA	New York Heart Association
PDCM	pediatric dilated cardiomyopathy
SV	stroke volume
